# Retrospective analysis of platelet rich plasma injections for ankle osteoarthritis in athletic population

**DOI:** 10.1186/s12891-026-09790-1

**Published:** 2026-04-13

**Authors:** Simone Natali, Iacono Venanzio, Daniele Screpis, Gianluca Piovan, Laura Mazzucco, Claudio Zorzi

**Affiliations:** 1 Department of Orthopaedics, IRCCS Ospedale Sacro Cuore Don Calabria, Viale Luigi Rizzardi, 4, Negrar di Valpolicella, Verona 37024 Italy; 2 Blood Component and Regenerative Medicine Laboratory, Alessandria Hospital, Alessandria, Italy

**Keywords:** Ankle osteoarthritis, Platelet-rich plasma (PRP), Intra-articular injection, Pain management, Return to sport

## Abstract

**Background:**

Osteoarthritis (OA) of the ankle, often post-traumatic in origin, is a common cause of pain and disability, particularly in young and athletic individuals. Platelet-rich plasma (PRP) has emerged as a valuable orthobiologic option due to its regenerative and anti-inflammatory properties. This study aimed to evaluate the efficacy of intra-articular PRP in patients with ankle OA, identify potential prognostic factors, and define the patient profile most likely to benefit, with a focus on athletes.

**Methods:**

This single-centre, real-world retrospective study assessed pain and function using Visual Analogue Scale (VAS) and American Orthopaedic Foot and Ankle Society (AOFAS) scores over a 36-month follow-up. PRP was prepared using a specific standardized method and administered in an outpatient setting. Outcomes were analyzed by OA grade, sex, and athletic status. Laboratory assays quantified platelet and leukocyte concentrations and interleukin-1 receptor antagonist (IL-1ra) release.

**Results:**

PRP treatment led to rapid and sustained improvements in both pain and function. The study included 178 subjects. Mean VAS scores decreased from 6.6 at baseline to 2.4 at 6 months and remained stable through 36 months. Mean AOFAS scores improved from 49.4 to 81.4 at 6 months and were maintained thereafter. Benefits were greatest in milder OA grades and in athletes, most of whom returned to sport. Age, sex, sporting status, and OA grade were significant predictors of functional outcomes at both 6 and 24 months. The overall failure rate was low (5.6%). Laboratory analysis demonstrated a 5.4-fold platelet enrichment, moderate leukocyte content, and a sixfold increase in IL-1ra levels, reflecting a cellular composition consistent with the anti-inflammatory property of the preparation.

**Conclusion:**

Intra-articular PRP prepared with a standardized method appeared to provide safe, sustained clinical benefits in ankle OA, particularly in younger and athletic patients with early-stage disease. A double-blind controlled trial will be necessary to further validate these findings.

**Supplementary Information:**

The online version contains supplementary material available at 10.1186/s12891-026-09790-1.

## Introduction

Osteoarthritis (OA) is a globally prevalent chronic degenerative disorder characterized by progressive anatomical and physiological alterations of joint tissues, leading to pain, stiffness, swelling, and impaired joint function [1, 2]. It is among the most common chronic health conditions worldwide [1]. Pathophysiologically, OA results from mechanical and biological processes that disrupt the balance between cartilage degradation and synthesis, as well as subchondral bone remodelling [3].

While OA most commonly affects the knee and hip, the ankle is less frequently involved. Although often underestimated, advanced ankle OA can be highly debilitating, with a quality-of-life burden comparable to that of hip or knee OA [4].

Primary ankle OA is relatively uncommon, with most cases being secondary to trauma [4]. Post-traumatic osteoarthritis (PTOA) represents the predominant aetiology, accounting for 70–78% of ankle OA cases. It is more common in younger and obese individuals [5, 6], and often results from sport-related trauma [7, 8]. Risk factors for OA include both person-level determinants - such as increasing age, female sex, altered joint biomechanics, genetic predisposition and adiposity- and joint-level factors, including previous injury, repetitive loading due to occupation or sport activities [9, 10], and malalignment [11].

The degenerative and inflammatory changes associated with OA impose a significant functional burden, particularly for individuals whose occupations or lifestyles require high physical performance. In athletes and physically active patients, the challenge is twofold: to alleviate pain while enabling a rapid and complete return to activity [7]. In this context, therapeutic approaches must not only manage symptoms but also support the biological repair of damaged tissues.

Management options for ankle OA include conservative measures, injection therapies, and surgery [12]. Current management is often conceptualized as a treatment pyramid, progressing from less invasive to more invasive strategies. Initial treatment typically consists of lifestyle modifications, physical therapy, footwear adaptations, orthotics, and pharmacological agents such as paracetamol or non-steroidal anti-inflammatory drugs, though their use remains debated due to variable efficacy and potential side effects [12]. In this perspective, delaying invasive procedures is a key goal. Injectable therapies therefore represent an intermediate option, with platelet-rich plasma (PRP) attracting growing interest for its minimally invasive nature, ability to promote cartilage repair and modulate inflammation through a minimally invasive approach [13].

First introduced in the 1970s, PRP has since been studied extensively across a wide range of clinical applications. It is derived from the patient’s own blood and contains a platelet concentration typically 3 to 5 times higher than physiological levels [14, 15]. Platelets, through their α-granules, release a variety of growth factors, cytokines, and proteins upon activation, creating a microenvironment conducive to tissue repair [7, 14]. Given its regenerative potential, PRP has been increasingly investigated as a therapeutic option for joint disorders, including ankle OA [13, 16–18]. By increasing the local platelet concentration at the site of injury, PRP may be particularly useful in chronic conditions unresponsive to conventional therapies, where healing is impaired. In such cases, PRP can provide both mechanical stimulation - by triggering a controlled inflammatory response - and biological stimulation through growth factor release, thereby enhancing tissue regeneration [19].

This retrospective, real-world study analysed data from 178 patients who received intra-articular PRP injections for the treatment of ankle osteoarthritis. The main objective was to identify potential risk factors and to define the patient profile most likely to benefit from this therapy. Particular attention was given to athletes. Laboratory analysis was also performed to determine the concentration of PRP component.

## Methods

### Patient population and outcomes

This is a retrospective, single-centre, real-world study, designed to evaluate the clinical effectiveness and risk factors of intra-articular PRP injections in patients with ankle OA. Inclusion criteria for data extraction from the institutional clinical registry were as follows: male and female patients diagnosed with ankle OA; provision of written informed consent; treatment with intra-articular PRP injection; availability of clinical records documenting ankle-related history and previous treatments; and documented outcomes of pain and function, routinely assessed at the centre using validated instruments, specifically the American Orthopaedic Foot and Ankle Society (AOFAS) score and the Visual Analog Scale (VAS). For patients with a history of sports activity, information on athletic status and return to sport had to be available. Furthermore, eligible patients were required to have at least one follow-up assessment at 6 months (T1), 12 months (T2), 24 months (T3) and 36 months (T4).

Patients who had received more than one PRP injection or presented with osteochondral lesions were excluded from the analysis. Obesity (BMI ≥ 30) was also considered an exclusion criterion, in accordance with clinical practice.

The primary outcome was the clinical effectiveness of intra-articular PRP therapy in improving pain and function in patients with ankle osteoarthritis. Efficacy was assessed at follow-up intervals (6, 12, 24 and 36 months) and stratified by athlete and non-athlete subgroups.

Clinical outcomes were measured using the two validated tools described above: the AOFAS, a composite measure evaluating ankle and hindfoot function through sections on pain, function, and alignment, offering a comprehensive assessment of joint status. Scores range from 0 to 100, with higher scores indicating better function and improvement [20, 21]; and the VAS, a unidimensional scale ranging from 1 (no pain) to 10 (worst possible pain) [22].

Secondary objectives included the identification and analysis of risk factors that might influence pain relief and functional recovery following PRP. Furthermore, laboratory tests were performed in collaboration with the Laboratory for Blood Component Preparation and Regenerative Medicine (Alessandria). PRP was characterized by assessing platelet and leukocyte concentrations (per mm³) and interleukin-1 receptor antagonist (IL-1ra) release (semi-quantitative assay, pg/mL).

### Treatment procedure

**Fig. 1:**
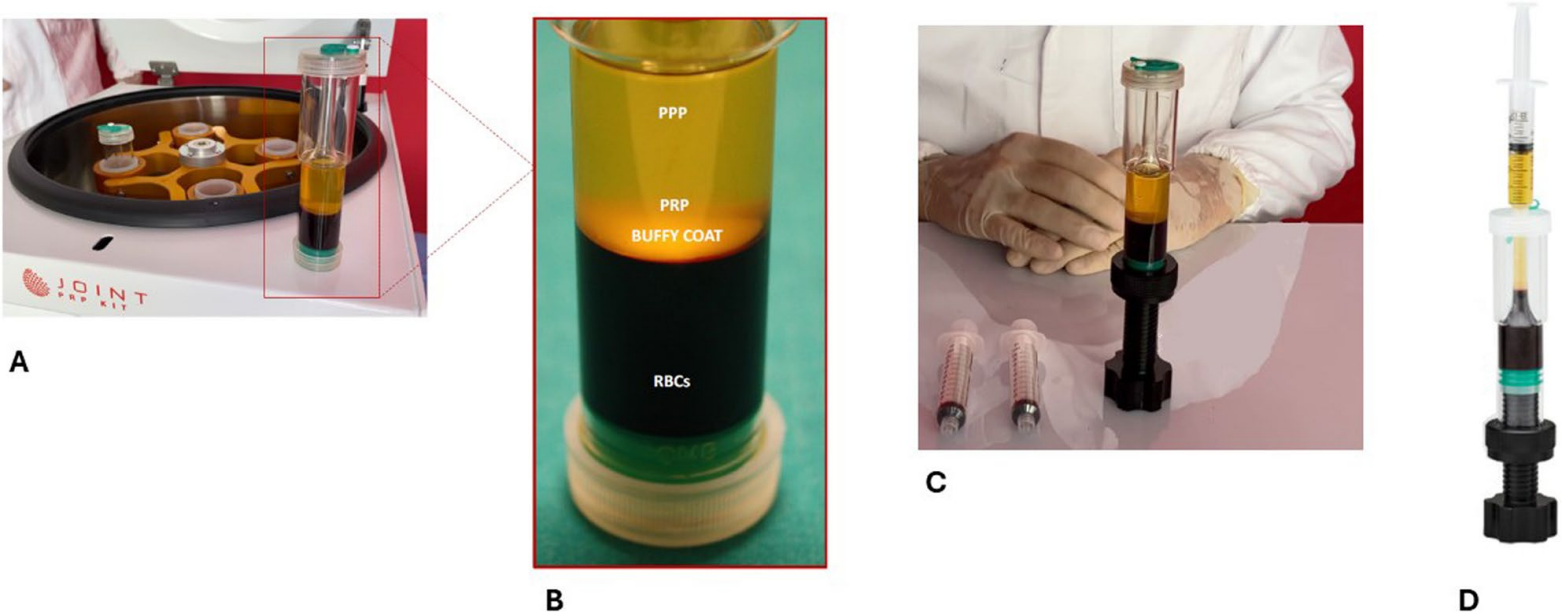
PRP preparation procedure. Blood samples are placed in a centrifuge for separation of components (**A**). After centrifugation, the tube shows distinct layers: PPP in the upper portion, PRP in the middle layer, a thin buffy coat containing leukocytes, and RBCs at the bottom (**B**). PRP is then extracted under sterile conditions and transferred into a syringe for intra-articular injection (**C**, **D**). PPP = platelet-poor plasma; PRP = platelet-rich plasma; Buffy coat = leukocyte-rich layer; RBCs = red blood cells

The JOINT PRP KIT (Joint S.r.l.), a sterile, single-use separation device designed for producing platelet concentrate from autologous peripheral blood, was employed to prepare Leuco-Rich PRP through a single 5-minute centrifugation cycle. Following clinical assessment to confirm eligibility, peripheral venous blood (20–30 mL) was drawn from each patient into a syringe preloaded with 2–3 mL of sodium citrate (3.8%) as anticoagulant. The blood sample was gently mixed and transferred into the separation device (Fig. 1A), which was then centrifuged using a preset program (3200–2800 rpm for 5 min). Following centrifugation, blood components were separated into red blood cells (RBCs), buffy coat, PRP, and platelet-poor plasma (PPP), as shown in Fig. 1B. PPP was first removed, and PRP was subsequently collected using a sterile extraction system (Fig. 1C and D). A final volume of approximately 2–3 mL of PRP was obtained per kit and used immediately after gentle homogenization. In cases of bilateral treatment, two kits were processed simultaneously to obtain a doubled PRP volume. No exogenous activation of PRP was performed prior to injection.

Intra-articular PRP injections were administered using an anteromedial approach, which constituted the intervention of interest in this study. Anatomical landmarks are identified by palpation, locating the site of infiltration. Local anesthetic was not used. Post-injection, patients were instructed to observe a brief period of strict protection (rest and ice) and to use analgesics as needed on the day of the procedure.

For all patients, PRP treatment was administered in an outpatient setting according to a standard protocol of one intra-articular infiltration every two weeks for a total of three infiltrations [23]. Follow-up assessments were conducted at regular intervals of 6, 12, and 24 months after the procedure, in accordance with clinical practice. Some patients were followed for up to 36 months. During these visits, physicians collected patient demographics, medical history, AOFAS and VAS scores, as well as information about treatments performed after PRP infiltration. Return to sporting activity and any treatment failure were considered clinically relevant outcomes and were recorded at the respective follow-up visits. Failure is defined as cases in which pain level and functionality remain unchanged or worsen compared to pre-treatment levels.

This retrospective, single-centre, uncontrolled, pilot study was conducted with the highest respect for individual participants. All patients provided their informed consent to the use of their medical records and personal data at the time of admission. The procedures followed were in accordance with the ethical standards of the responsible committee on human experimentation (institutional and national) and with the revision of the Declaration of Helsinki, 2024. The study protocol was reviewed and approved by the Ethics Committee of the Clinical Research Unit, IRCCS Sacro Cuore Don Calabria Hospital, Verona (protocol no. 61386, approved on 19 September 2018).

### Statistical methods

Patient characteristics were summarised using counts and percentages for categorical variables and means with standard deviation and 25th-75th percentile for continuous variables. These summaries were also stratified according to different risk factors and PRP treatment failure. VAS and AOFAS scores were summarised by time point (baseline, 6-month, 12-month, 24-month, and 36 months). For continuous variables, mean trends over time were plotted, stratified by risk factors. The assumption of normality for continuous variables was assessed using the Shapiro–Wilk test. Differences between subgroups were evaluated using the chi-square test or Fisher’s exact test for categorical variables, and the unpaired t-test or the Wilcoxon rank-sum test for continuous variables, as appropriate. Linear regression models were also used to evaluate changes in VAS and AOFAS scores from baseline to the 6-month and 24-month visits, adjusting for baseline values. The regression estimates for the OA score are based solely on patients for whom data was available. All tests were two-sided, and a p-value < 0.05 was considered statistically significant. All analyses were performed using SAS version 9.4 (SAS Institute Inc., Cary, NC, USA).

## Results

A total of 178 subjects were enrolled, 86 females (48.3%) and 92 males (51.7%), aged between 14 and 85 years (mean 53.01 ± 15.21; median 55.5 years; IQR 44–64), all presenting with traumatic or inflammatory ankle osteoarthritis. Each participant received an intra-articular PRP injection via an anteromedial approach. 32% of the patients reported being athletes (data collected at baseline). A wide range of sports was represented: 14 patients (25%) practiced football, 9 (16%) running, 8 (14%) trekking or climbing, 7 (12%) cycling, and 6 (11%) athletics. Gym training, volleyball, and pilates/yoga were each reported by 3 patients (5%), while dancing was reported by 2 patients (4%), and swimming and tennis by 1 patient each (2%). OA severity at baseline was grade I in 40%, grade II in 32% and grade III in 18%; the remaining patients had unclassified or missing grade information. Baseline characteristics are detailed in Table 1.

**Table 1:** Patient demographics

	*N* (%)
Sex	
Female	86 (48.31)
Male	92 (51.69)
Ankle treated	
Left	89 (50.00)
Right	70 (39.33)
Both	19 (10.67)
Osteoarthritis grade	
1	72 (40.45)
2	57 (32.02)
3	33 (18.54)
NA	16 (8.99)
Sportmen	
Yes	57 (32.02)
No	121 (67.98)
Previous treatment	
Yes	27 (15.17)
No	151 (84.83)

At baseline, a statistically significant sex difference was observed across OA severity groups (*p* = 0.004), with a progressive increase in the proportion of female patients in the more severe categories. Age likewise showed a significant upward trend with increasing OA severity (*p* = 0.002). No significant differences were found in the side of the ankle treated (*p* = 0.78) or history of previous treatments (*p* = 0.32). Athlete distribution was also non-homogeneous across OA grades, with the majority presenting with grade I OA (Table 2).

**Table 2:** Patient’s characteristic comparison by osteoarthritis grade

	1 (*N* = 72)	2 (*N* = 57)	3 (*N* = 33)	*P*-value
Sex, female	26	31	23	**0.004**
Ankle treated				0.78
Left	36 (50.00)	24 (42.11)	18 (54.5)	
Right	29 (40.28)	25 (43.86)	12 (36.36)	
Both	7 (9.72)	8 (14.04)	3 (9.09)	
Previous treatment	11 (15.28)	7 (12.28)	8 (24.24)	0.32
Sportmen	36 (50.00)	12 (21.05)	6 (18.18)	**0.0003**
Age (years)	48.43 ± 15.32; 50.00 (38.50–60.00)	54.72 ± 14.79; 56.00 (49.00–64.00)	58.79 ± 13.46; 58.00 (52.00–68.00)	**0.002**

Values are presented as N(%), with percentages calculated on the total number of patients. Age is presented as mean ± standard deviation (SD) and as median (25th–75th percentile). Values in bold (*P*-value) were statistically significant (*P* < 0.05)

**Fig. 2:**
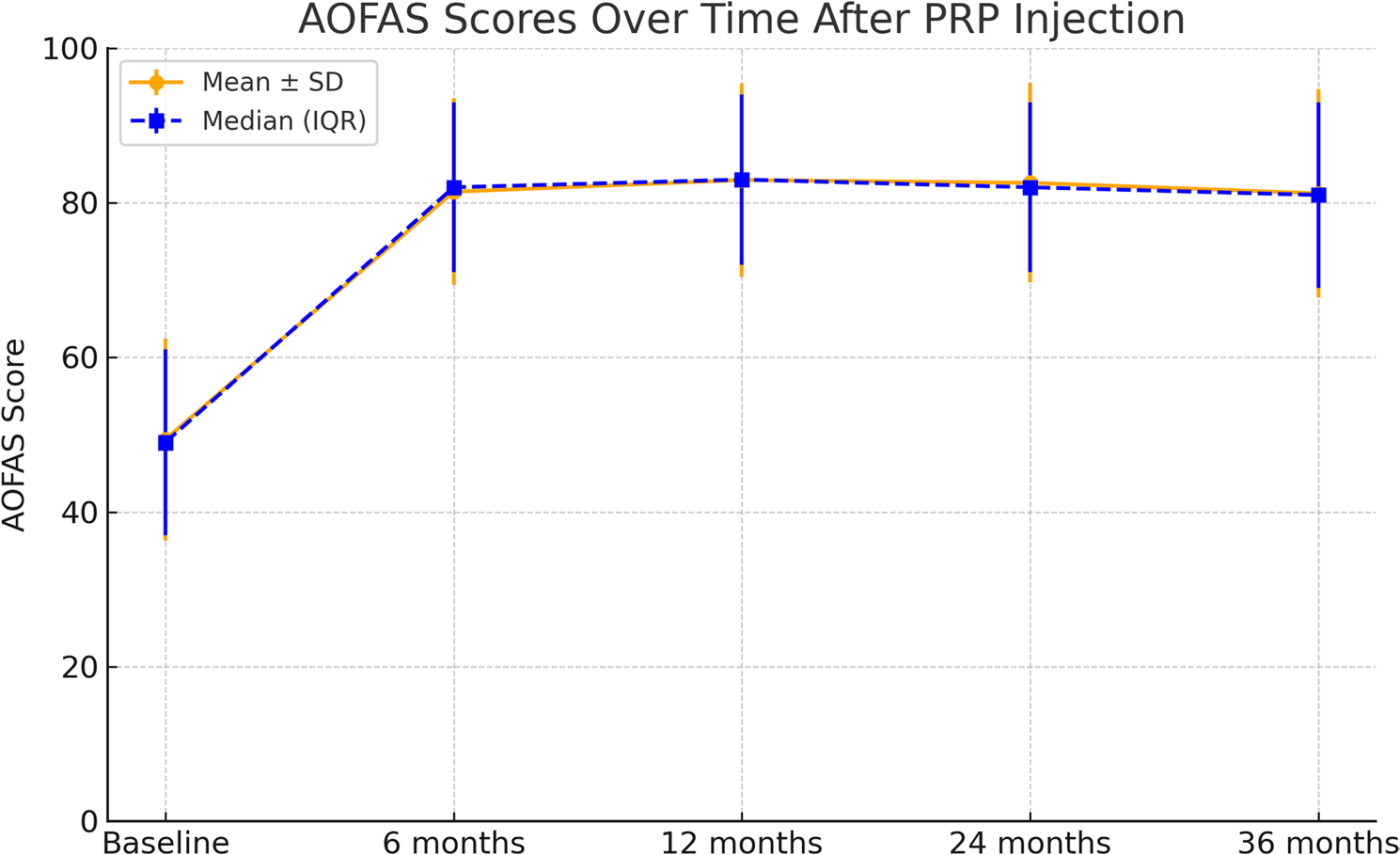
Mean and median AOFAS score values over time

**Fig. 3:**
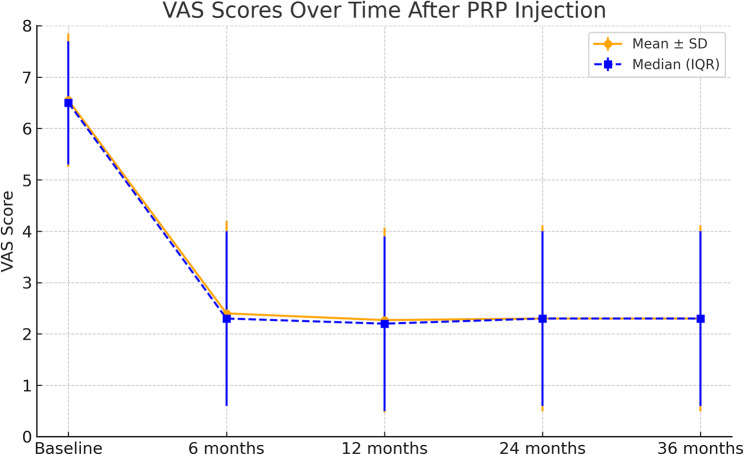
Mean and median VAS score values over time

Overall, the mean VAS score decreased from 6.55 ± 1.30 at baseline to 2.40 ± 1.80 at 6 months (*p* < 0.001), 2.27 ± 1.80 at 12 months, 2.30 ± 1.81 at 24 months, and 2.30 ± 1.81 at 36 months, indicating a substantial and sustained improvement over time (Fig. 2). The mean AOFAS score improved significantly from 49.37 ± 13.10 at baseline to 81.42 ± 12.09 at 6 months (*p* < 0.0001), with further stabilization at 12 (82.94 ± 12.52), 24 (82.58 ± 12.93), and 36 months (81.21 ± 13.50), showing a sustained functional benefit throughout the follow-up period (Fig. 3).

**Fig. 4:**
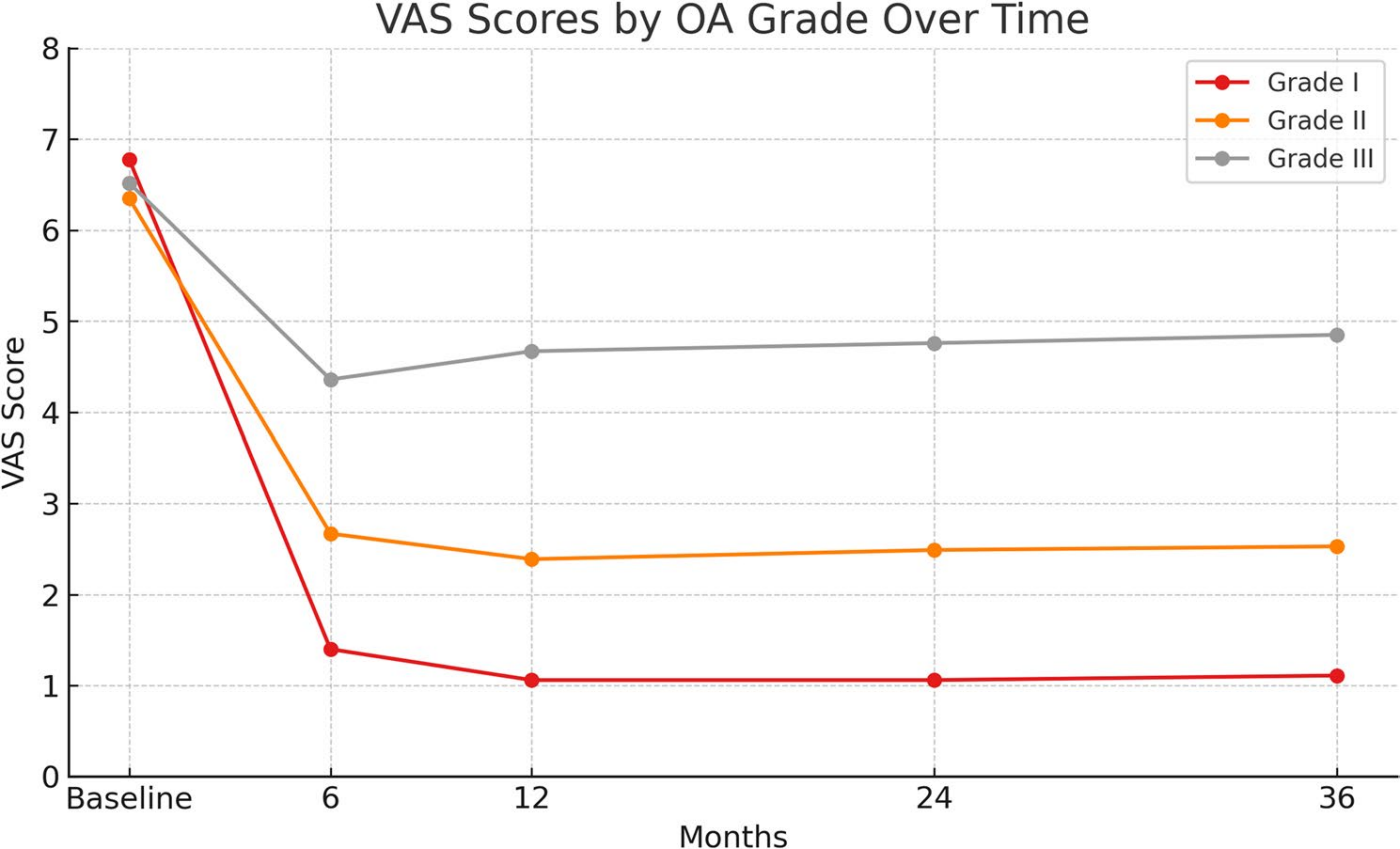
VAS score over time by OA grade

**Fig. 5:**
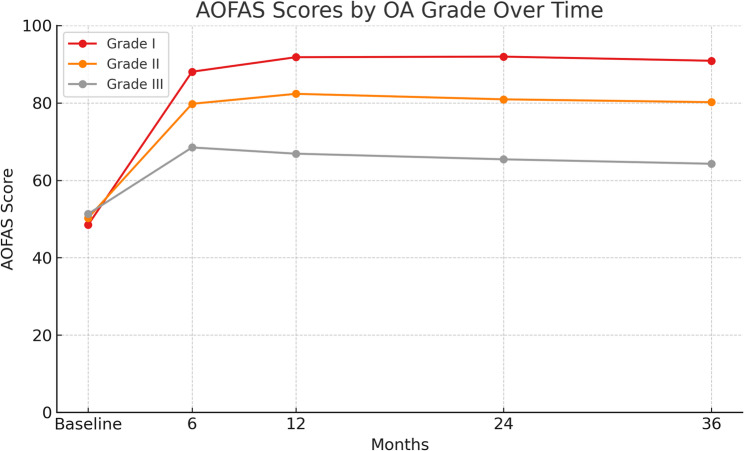
AOFAS score over time by OA grade

Analysis of pain and functional scores by OA grade confirmed a marked reduction at 6-month for all grades, sustained through 24 and 36 months (all *p* < 0.0001). Specifically, VAS score improved from 6.78 to 1.40 in grade I, 6.35 to 2.67 in grade II, and 6.52 to 4.36 in grade III. These improvements remained stable through 12, 24, and 36 months, although grade III patients showed a slight increase at 36 months (4.85), indicating a plateau at a higher pain level (Fig. 4, Table S1 Supplementary Appendix). Similarly, AOFAS scores improved significantly (*p* < 0.0001) from 48.50 to 88.07 in grade I, 50.28 to 79.77 in grade II, and 51.30 to 68.48 in grade III at 6 months. Scores remained stable over time, with grades I and II maintaining high values, while grade III declined slightly at 36 months (64.27), still representing a clinically relevant benefit compared with baseline (Fig. 5, Table S2 Supplementary Appendix).

A total of ten failures were recorded (5.6%). No statistically significant difference was observed in the rate of occurrence between male and female subjects.

Linear regression analysis showed that, for VAS outcomes, sex and OA grade were significant risk factors at both 6 and 24 months, whereas age and sporting status were significant only at 24 months (Tables S3–S4, Supplementary Appendix). For AOFAS outcomes, age, sex, sporting status, and OA grade were significant predictors at both 6 and 24 months (Tables S3–S4, Supplementary Appendix). Women consistently reported higher VAS scores and lower AOFAS scores than men across all follow-up visits, with differences already evident at 6 months (*p* = 0.002 and *p* = 0.02, respectively), suggesting an overall less favorable clinical course in female patients. Notably, the treated ankle side and previous treatments were not identified as risk factors for either outcome (Tables S3–S4, Supplementary Appendix).

### Impact of sporting activity on outcomes

Physically active individuals represented 32% of the cohort (57/178), including 20 women and 37 men. Among them, 14 (25%) practiced soccer, 9 (16%) running, 8 (14%) mountaineering (including trekking and climbing), 7 (12%) cycling, and smaller proportions were engaged in athletics, dance, gym training, volleyball, pilates/yoga, swimming, or tennis. OA grade distribution differed significantly between groups (*p* = 0.0004), with sportive patients presenting lower OA grades at baseline. No significant differences in baseline VAS or AOFAS scores were observed between athletes and non-athletes (*p* = 0.69 and *p* = 0.34, respectively). Treatment failures did not differ significantly between patients engaged in regular sports activity versus those who are not (*p* = 0.17); however, they occurred in only 1 of 57 athletes (1.7%) compared with 9 of 121 non-athletes (7.4%).

**Fig. 6:**
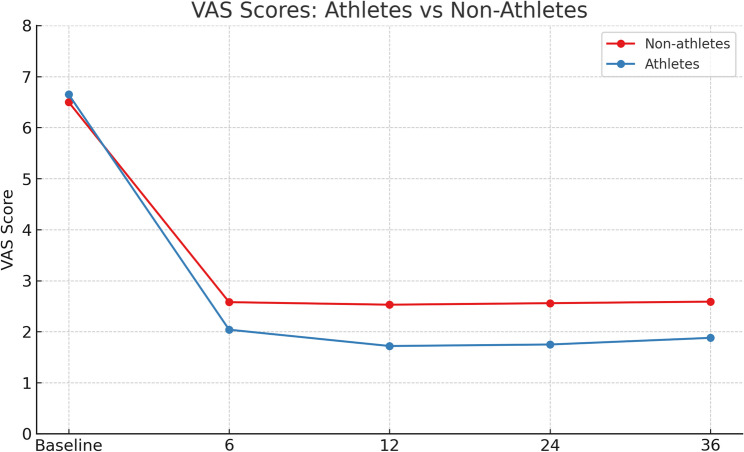
VAS score over time by sports-active population

**Fig. 7:**
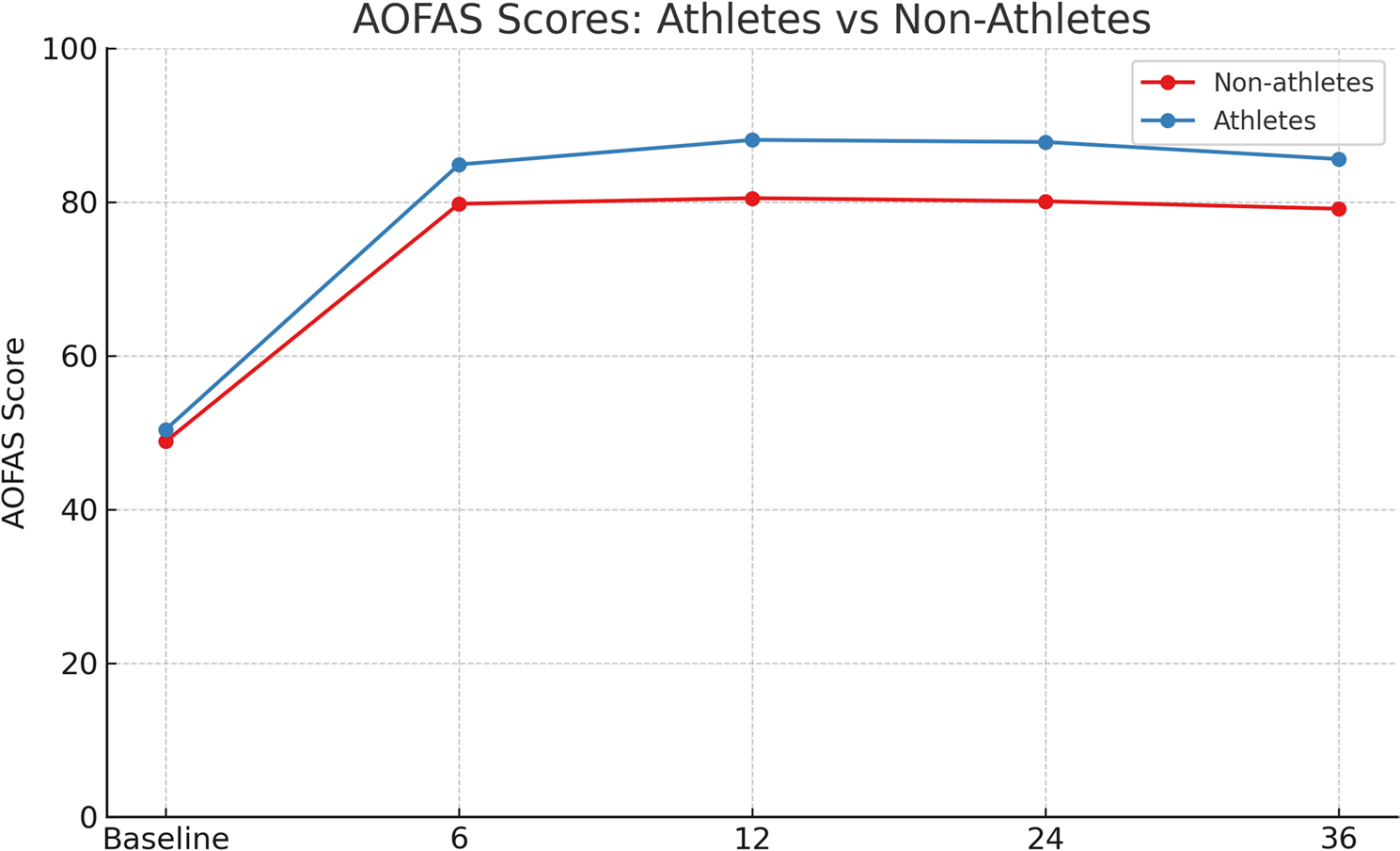
AOFAS score over time by sports-active population

In sports-active subjects, VAS score reduction was significantly greater at 6 months compared with non-sportive patients (*p* = 0.03), and this difference persisted at 24 months [median VAS 2.00 (1.00–4.00) vs. 1.00 (1.00–2.00); *p* = 0.004] and at 36 months (*p* = 0.008) (Fig. 6). All changes from baseline were statistically significant (Table S5 Supplementary Appendix). Active subjects also demonstrated significantly better mobility at 6 months (*p* = 0.002), a difference that persisted at 24 months [median AOFAS 91.00 (81.00–97.00) vs. 83.00 (71.00–91.00); *p* = 0.0001; Fig. 7, Table S6 Supplementary Appendix] and beyond, although relative changes from baseline within each group did not differ significantly. Patients who resumed sports after treatment showed significantly greater improvement in AOFAS scores at all follow-up visits compared to baseline (Table S7 Supplementary Appendix). Among the 57 patients who were active before treatment, 48 returned to sport following PRP, while 6 additional previously non-active patients initiated sporting activity after treatment. Overall, 54 patients (30.3% of the cohort) were engaged in sport post-PRP, and they reported lower pain scores at all follow-up visits (all *p* < 0.001; Table S8 Supplementary Appendix). More men than women resumed sporting activity (35, 38.0% vs. 19, 22.1%; *p* = 0.02).

### Laboratory characterization of PRP

Laboratory characterization demonstrated a mean platelet concentration 5.4-fold higher than baseline. Platelets were easily and rapidly recovered from the buffy coat obtained after centrifugation, which also resulted in leukocyte collection (mean 13.7 × 10³/mm³; 2.4-fold higher than baseline) with a predominance of mononuclear cells (61% lymphocytes/monocytes). This leukocyte enrichment was associated with a substantial release of IL-1ra, with mean concentrations at least sixfold higher than plasma values (3,514–4,075 pg/mL vs. 401–596 pg/mL in plasma).

## Discussion

PTOA is the most common form of ankle OA [24], arising from the limited regenerative capacity of hyaline cartilage and the increased contact stresses following trauma, particularly prevalent among athletes [7, 8]. In this setting, orthobiologics have gained increasing attention for their potential to promote tissue repair with a minimally invasive profile. PRP is one such therapy [13, 25, 26].

This study provides robust real-world evidence supporting the use of PRP in ankle OA. Data from 178 patients were analysed over a follow-up period of 36 months, demonstrating rapid and sustained improvements in both pain and function, particularly among athletic individuals. Within the first 6 months, patients experienced a marked reduction in pain, as reflected by the decrease in mean VAS scores, and parallel gains in AOFAS score. These benefits remained stable thereafter, indicating that the early therapeutic effect is maintained over time. Notably, younger age, male sex, lower OA grade, and sport activity were associated with greater improvements in VAS and AOFAS scores over time.

This pattern of sharp initial improvement followed by a plateau is consistent with the biological rationale of PRP therapy, in which the release of growth factors promotes an acute phase of tissue repair and rapid symptom relief that is subsequently stabilized. The regenerative potential of PRP depends on platelet and leukocyte concentrations as well as on the presence of IL-1ra, which exerts anti-inflammatory effects by binding IL-1, one of the most abundant pro-inflammatory cytokines [27, 28]. Its concentration may vary depending on the PRP preparation method. Laboratory tests demonstrated that the procedure involved in this study yields platelet and leukocyte concentrations approximately 5.4-fold and 2.4-fold higher than baseline, respectively, as well as IL-1ra levels six times higher than those measured in plasma (internal, unpublished data). These characteristics may contribute synergistically to the anti-inflammatory and regenerative potential of the final PRP product, thereby validating the clinical outcomes of the treatment.

The results are consistent with previous studies reporting beneficial effects of PRP on both pain and functional outcomes [16, 18, 29]. However, heterogeneity in PRP preparation protocols, dosing regimens, and follow-up durations across studies may limit direct comparability and complicate the definition of an optimal patient profile. Furthermore, literature data on ankle OA and conservative treatment are scarce and yielding mixed results. This is also due to its comparatively lower prevalence in relation to OA affecting other joints. Importantly, Paget et al. conducted a randomised placebo-controlled trial in which patients with ankle OA were administered either a PRP injection or a placebo. The authors found no significant difference between the two groups in terms of symptom relief or functionality over 26 weeks follow-up [30]. This highlights the ongoing uncertainty regarding the clinical effectiveness of PRP in this setting and underscores the need for cautious interpretation of non-controlled studies, including the present one. Differences in PRP formulation, patient selection, disease severity, and treatment protocols may partially explain these contrasting findings and should be considered when comparing results across studies.

In the present study, particular attention was given to physically active individuals, allowing for a more detailed assessment of outcomes in this subgroup. Sport-related ankle injuries are common and represent a risk factor for developing OA [9, 10]. Subgroup analyses offered valuable insights into patient profiles associated with the greatest benefit. In our cohort, 32% of participants were individuals engaged in regular physical activity at baseline, and most resumed sport activity following treatment. A variety of sports was represented, with the majority involving endurance disciplines with high joint impact, such as football, running, cycling, and climbing. Despite similar baseline scores, athletes experienced greater reduction in pain and better functional outcomes compared with non-athletes, and return to sport was associated with improved VAS and AOFAS scores regardless of prior athletic status. These findings suggest that PRP facilitates not only symptom relief but also functional recovery sufficient to enable resumption of sport. Supporting evidence comes from a pilot study in rugby players with syndesmosis injuries, in which PRP treatment enabled significantly earlier return to play compared to conservative management [31].

Sex-related differences were also observed: women reported higher pain and lower function across all follow-up visits and showed lower rates of return to sport. These disparities may partly reflect the higher OA grade seen in women at baseline, but sex-based differences in pain perception, inflammatory response, and cartilage biology have also been reported [32].

Baseline OA grade significantly influenced outcomes, with patients with grade I–II disease achieving the most pronounced and sustained improvements. Patients with grade III OA also benefited, but to a lesser extent, and showed a gradual decline over time, consistent with previous reports of lower PRP efficacy in late-stage OA [23, 33]. These results highlight the importance of considering disease stage when selecting candidates for treatment and setting realistic expectations.

Importantly, previous nonsurgical treatments did not significantly affect outcomes, suggesting that PRP remains effective even in patients who have failed other conservative therapies and may serve as a valuable adjunct for those with more advanced disease who require additional therapeutic options.

Among the key strengths of this study are its relatively large sample size, extended follow-up (up to 36 months), and detailed subgroup analyses, which allowed identification of clinical predictors of success. While the overall population benefited from treatment, younger age, male sex, lower OA grade, and sport activity emerged as predictors of the most pronounced benefits. These findings may help refine patient counselling and personalize treatment strategies without limiting the indication to selected subgroups.

Nonetheless, some limitations must be acknowledged. The retrospective design limits the ability to control for all confounding variables, and the absence of a control group precludes direct comparison with alternative treatments. Moreover, some potentially relevant variables (such as limb alignment, prior ankle surgeries, duration of symptoms, and post-injection rehabilitation strategies) were not consistently available for all patients and could therefore not be systematically analysed. Also, there was no distinction between different type of athletes among the physically active individuals. Prospective controlled studies are warranted to better evaluate long-term effects and confirm these findings. Despite these limitations, the study reflects real-world practice in outpatient settings, enhancing its external validity and clinical relevance.

## Conclusion

This study on 178 patients suggest that PRP prepared with this specific system is associated with improvements in pain and function in patients with ankle OA. Sustained clinical benefits and a low rate of treatment failure were observed over time, particularly in early and moderate stages of disease and among physically active individuals. A high proportion of patients returned to sport, with some initiating new sporting activities after treatment, suggesting a potential role of PRP in supporting functional recovery and an active lifestyle. These findings may be related to the biological profile of PRP obtained with this specific procedure, characterized by high concentrations of platelets, leukocytes, and IL-1ra. Age, sex, OA grade, and sports participation emerged as key predictors of a favourable response. However, given the retrospective and uncontrolled design, these findings should be interpreted with caution, and further controlled studies are warranted to confirm these observations.

## Supplementary Information

Supplementary Material 1.

## Data Availability

The data that support the findings of this study are available from the corresponding author upon reasonable request.

## Abbreviations

OA: Osteoarthritis
PRP: Platelet-rich plasma
VAS: Visual analogue scale
AOFAS: American orthopaedic foot and ankle society
IL-1ra: Interleukin-1 receptor antagonist
PTOA: Post-traumatic osteoarthritis
LR-PRP: Leuco-rich platelet-rich plasma
RBCs: Red blood cells
PPP: Platelet-poor plasma
SD: Standard deviation
IQR: Interquartile range
