# Supplementary material for: Retrospective analysis of platelet rich plasma injections for ankle osteoarthritis in athletic population
Source: BMC Musculoskelet Disord. 2026 Apr 13;27:559. doi: 10.1186/s12891-026-09790-1 (PMC13326289; doi:10.1186/s12891-026-09790-1)
Supplement: Supplementary file 1 — Supplementary Material 1. [file 12891_2026_9790_MOESM1_ESM.docx]

**Supplementary data**

***Table S1.*** *VAS score comparison by OA grade*

|  | 1 (N=72) | 2 (N=57) | 3 (N=33) | P-value |
| --- | --- | --- | --- | --- |
| VAS Baseline | 6.78±1.31;7.00 (6.00-8.00) | 6.35±1.19;7.00 (5.00-7.00) | 6.52±1.33;6.00 (6.00-7.00) | 0.16 |
| VAS 6 month | 1.40±1.34;1.00 (0.00-2.00) | 2.67±1.24;3.00 (2.00-3.00) | 4.36±1.78;4.00 (3.00-5.00) | **<0.0001** |
| VAS 12 month | 1.06±1.06;1.00 (0.00-1.50) | 2.39±1.26;2.00 (2.00-3.00) | 4.67±1.73;5.00 (3.00-6.00) | **<0.0001** |
| VAS 24 month | 1.06±0.84;1.00 (0.50-1.50) | 2.49±1.02;2.00 (2.00-3.00) | 4.76±1.80;5.00 (3.00-6.00) | **<0.0001** |
| VAS 36 month | 1.11±0.85;1.00 (1.00-2.00) | 2.53±0.95;2.00 (2.00-3.00) | 4.85±1.62;5.00 (4.00-6.00) | **<0.0001** |

Mean±std; Median (25^th^-75^th^ percentile)

***Table S2.*** *AOFAS score comparison by OA grade*

|  | 1 (N=72) | 2 (N=57) | 3 (N=33) | P-value |
| --- | --- | --- | --- | --- |
| AOFAS Baseline | 48.50±13.33;46.00 (40.00-58.00) | 50.28±14.09;52.00 (43.00-60.00) | 51.30±12.58;57.00 (43.00-59.00) | 0.46 |
| AOFAS 6 month | 88.07±9.93;91.00 (84.00-97.00) | 79.77±8.25;81.00 (77.00-84.00) | 68.48±11.94;71.00 (59.00-77.00) | **<0.0001** |
| AOFAS 12 month | 91.82±7.63;92.00 (88.50-97.00) | 82.35±7.21;83.00 (77.00-87.00) | 66.88±11.62;69.00 (58.00-72.00) | **<0.0001** |
| AOFAS 24 month | 91.97±6.19;92.00 (87.00-97.00) | 80.93±7.88;81.00 (77.00-84.00) | 65.42±12.06;65.00 (57.00-72.00) | **<0.0001** |
| AOFAS 36 month | 90.90±8.73;91.00 (84.00-97.00) | 80.19±6.99;81.00 (77.00-84.00) | 64.27±11.80;65.00 (57.00-72.00) | **<0.0001** |

Mean±std; Median (25^th^-75^th^ percentile)

***Table S3.*** *Assessment of risk factors associated with variation between baseline and 6 months and baseline and 24 months of VAS score (p-value)*

|  | Baseline – 6 months | | Baseline 24 months | |
| --- | --- | --- | --- | --- |
|  | **LS means (SE)** | **p-value** | **LS means** | **p-value** |
| AGE | Not applicable | 0.09 | Not applicable | **0.006** |
| GENDER |  | **0.003** |  | **0.002** |
| FEMALE | -3.73 (0.19) |  | -3.81 (0.19) |  |
| MALE | -4.53 (0.18) |  | -4.65 (0.18) |  |
| ANKEL TREATED |  | 0.96 |  | 0.83 |
| LEFT | -4.11 (0.19) |  | -4.16 (0.19) |  |
| RIGHT | -4.16 (0.22) |  | -4.16 (0.19) |  |
| BOTH | -4.23 (0.41) |  | -4.34 (0.41) |  |
| PREVIOUS TREATMENT |  | 0.41 |  | 0.13 |
| YES | -3.89 (0.34) |  | -4.79 (0.23) |  |
| NO | -4.19 (0.15) |  | -4.00 (0.16) |  |
| SPORTMEN |  | 0.06 |  | **0.005** |
| YES | -4.52 (0.23) |  | -4.79 (0.23) |  |
| NO | -3.97 (0.16) |  | -4.00 (0.16) |  |
| OSTEOARTHRITIS GRADE |  | **<0.0001** |  | **<0.0001** |
| 1 | -5.19 (0.16) |  | -5.52 (0.14) |  |
| 2 | -3.88 (0.19) |  | -4.08 (0.15) |  |
| 3 | -2.20 (0.24) |  | -1.82 (0.20) |  |

Least squares mean (standard error)

***Table S4.*** *Assessment of risk factors associated with variation between baseline and 6 months and baseline and 24 months of AOFAS score (p-value)*

|  | Baseline – 6 months | | Baseline 24 months | |
| --- | --- | --- | --- | --- |
|  | **LS means (SE)** | **p-value** | **LS means** | **p-value** |
| AGE | Not applicable | **0.008** | Not applicable | **<0.0001** |
| SEX |  | **0.01** |  | **0.01** |
| FEMALE | 29.74 (1.28) |  | 30.64 (1.37) |  |
| MALE | 32.21 (1.23) |  | 35.62 (1.32) |  |
| ANKEL TREATED |  | 0.74 |  | 0.63 |
| LEFT | 32.33 (1.28) |  | 33.50 (1.55) |  |
| RIGHT | 31.30 (1.45) |  | 35.53 (2.95) |  |
| BOTH | 33.52 (2.75) |  | 32.49 (1.37) |  |
| PREVIOUS TREATMENT |  | 0.18 |  | 0.14 |
| YES | 29.20 (2.30) |  | 29.82 (2.47) |  |
| NO | 32.56 (0.97) |  | 33.82 (1.04) |  |
| SPORTMEN |  | **0.008** |  | **0.0001** |
| YES | 35.47 (1.56) |  | 38.49 (1.64) |  |
| NO | 30.44 (1.07) |  | 30.73 (1.13) |  |
| OSTEOARTHRITIS GRADE |  | **<0.0001** |  | **<0.0001** |
| 1 | 38.52 (1.13) |  | 42.34 (0.96) |  |
| 2 | 30.00 (1.27) |  | 31.20 (1.08) |  |
| 3 | 18.58 (1.67) |  | 15.63 (1.42) |  |

Least squares mean (standard error)

***Table S5.*** *VAS score comparison by sport activity*

|  | No sportmen (N=121) | Sportmen (N=57) | P-value |
| --- | --- | --- | --- |
| VAS Baseline | 6.50±1.23;7.00 (6.00-7.00) | 6.65±1.45;6.00 (5.00-8.00) | 0.69 |
| VAS 6 month | 2.58±1.75;2.00 (2.00-3.00) | 2.04±1.86;2.00 (1.00-3.00) | **0.03** |
| VAS 12 month | 2.53±1.87;2.00 (1.00-4.00) | 1.72±1.68;1.00 (1.00-2.00) | **0.003** |
| VAS 24 month | 2.56±1.87;2.00 (1.00-4.00) | 1.75±1.53;1.00 (1.00-2.00) | **0.004** |
| VAS >24 month | 2.59±1.81;2.00 (1.00-4.00) | 1.88±1.54;2.00 (1.00-3.00) | **0.008** |

Mean±std; Median (25^th^-75^th^ percentile)

***Table S6.*** *AOFAS score comparison by sport activity*

|  | No sportmen (N=121) | Sportmen (N=57) | P-value |
| --- | --- | --- | --- |
| AOFAS Baseline | 48.87±12.02;48.00 (43.00-57.00) | 50.42±15.20;57.00 (40.00-61.00) | 0.34 |
| AOFAS 6 month | 79.78±11.92;81.00 (72.00-87.00) | 84.89±11.82;87.00 (78.00-93.00) | **0.002** |
| AOFAS 12 month | 80.51±12.69;83.00 (72.00-91.00) | 88.09±10.50;91.00 (84.00-97.00) | **<0.0001** |
| AOFAS 24 month | 80.11±13.44;83.00 (71.00-91.00) | 87.82±10.00;91.00 (81.00-97.00) | **0.0001** |
| AOFAS >24 month | 79.13±13.74;81.00 (71.00-87.00) | 85.60±11.96;87.00 (78.00-97.00) | **0.001** |

Mean±std; Median (25^th^-75^th^ percentile)

***Table S7.*** *AOFAS score comparison by sport resumption*

|  | No resumption sport (N=124) | Resumption sport (N=54) | P-value |
| --- | --- | --- | --- |
| AOFAS Baseline | 49.69±11.97;52.00 (43.00-58.00) | 48.63±15.48;46.00 (35.00-60.00) | 0.70 |
| AOFAS 6 month | 79.22±11.99;81.00 (71.00-87.00) | 86.46±10.83;88.50 (81.00-97.00) | **<0.0001** |
| AOFAS 12 month | 80.19±12.74;83.00 (71.00-91.00) | 89.24±9.39;91.00 (84.00-97.00) | **<0.0001** |
| AOFAS 24 month | 79.78±13.36;83.00 (71.00-91.00) | 89.00±9.15;91.00 (84.00-97.00) | **<0.0001** |
| AOFAS >24 month | 78.85±13.70;81.00 (71.00-87.00) | 86.57±11.44;88.00 (78.00-97.00) | **0.0001** |

Mean±std; Median (25^th^-75^th^ percentile)

***Table S8.*** *VAS score comparison by sport resumption*

|  | No resumption sport (N=124) | Resumption sport (N=54) | P-value |
| --- | --- | --- | --- |
| VAS Baseline | 6.51±1.25;7.00 (5.50-7.00) | 6.65±1.42;6.00 (5.00-8.00) | 0.65 |
| VAS 6 month | 2.69±1.79;2.00 (2.00-3.00) | 1.76±1.66;1.00 (0.00-3.00) | **0.0005** |
| VAS 12 month | 2.62±1.89;2.00 (1.00-4.00) | 1.46±1.45;1.00 (0.00-2.00) | **<0.0001** |
| VAS 24 month | 2.64±1.88;2.00 (1.00-4.00) | 1.54±1.36;1.00 (1.00-2.00) | **0.0001** |
| VAS >24 month | 2.65±1.83;2.00 (1.00-4.00) | 1.70±1.37;1.50 (1.00-2.00) | **0.0007** |

Mean±std; Median (25^th^-75^th^ percentile)
